# Cholesterol Synthesis Is Associated with Hepatic Lipid Content and Dependent on Fructose/Glucose Intake in Healthy Humans

**DOI:** 10.1155/2012/361863

**Published:** 2011-11-29

**Authors:** Guenther Silbernagel, Dieter Lütjohann, Juergen Machann, Sabrina Meichsner, Konstantinos Kantartzis, Fritz Schick, Hans-Ulrich Häring, Norbert Stefan, Andreas Fritsche

**Affiliations:** ^1^Division of Endocrinology, Department of Internal Medicine, Diabetology, Nephrology, Vascular Disease, and Clinical Chemistry, Eberhard-Karls-University Tübingen, Otfried-Müller-Straße 10, 72076 Tübingen, Germany; ^2^Institute of Clinical Chemistry and Clinical Pharmacology, University Clinic Bonn, Sigmund-Freud-Straße 25, 53127 Bonn, Germany; ^3^Section on Experimental Radiology, Department of Diagnostic Radiology, Eberhard-Karls-University Tübingen, Hoppe-Seyler-Straße 3, 72076 Tübingen, Germany

## Abstract

Visceral obesity and fatty liver have been related to high synthesis and low absorption of cholesterol. This study aimed to investigate the associations of cholesterol metabolism with liver and visceral fat content in healthy humans. Another objective was to explore the effects of very-high-fructose and very-high-glucose diets on cholesterol homeostasis. We report on a cohort of 20 people (12 males, 8 females; age 30.5 ± 2.0 years; body mass index 25.9 ± 0.5 kg/m^2^) who completed a four-week dietary intervention study. Between the baseline and the followup examination the study participants in addition to a balanced weight-maintaining diet received 150 g of either fructose or glucose per day. Visceral and liver fat were measured with magnetic resonance (MR) imaging and ^1^H-MR spectroscopy, respectively. Cholesterol absorption and synthesis were estimated from the serum noncholesterol sterol concentrations. Performing cross-sectional analyses the lanosterol and desmosterol to cholesterol ratios were positively correlated with visceral and liver fat content (all *P* < .03). The lathosterol to cholesterol ratio decreased in response to high-fructose diet (*P* = .006) but not in response to high-glucose diet. To conclude, visceral and liver fat content are associated with cholesterol synthesis in healthy humans. Furthermore, cholesterol synthesis appears to be dependent on fructose/glucose intake.

## 1. Introduction

Serum cholesterol is either derived from intestinal absorption or from endogenous synthesis [1]. The individual balance of cholesterol absorption and synthesis is highly heritable [2]. The ATP-binding cassette transporters G5 and G8 (ABCG5/8) and the Niemann-Pick C1 Like1 protein (NPC1L1) play important roles in cholesterol homeostasis. Both genes encode proteins that are expressed in the intestine and regulate cholesterol absorption [3–5]. However, cholesterol absorption and synthesis are not only determined by genetic factors but also by the metabolic state [6–10]. For example, subjects with high body mass index display high synthesis and low absorption of cholesterol [6–8]. Furthermore, cholesterol synthesis prevails over cholesterol absorption in insulin resistance and type 2 diabetes [7–11]. In agreement, visceral obesity is associated with a high synthesis phenotype [12, 13]. Recently, fatty liver, which is thought to be involved in the pathogenesis of the metabolic syndrome [14–17], was also found to be associated with high cholesterol synthesis and low cholesterol absorption [18]. 

The present work aimed to investigate whether visceral and liver fat contents are correlated with cholesterol homeostasis in healthy humans. Our hypothesis was that even modest differences of liver and visceral fat content would be reflected by differences in cholesterol synthesis and absorption. To answer this question, we performed cross-sectional analyses in 20 healthy individuals who participated in a four-week dietary intervention (either very-high-fructose or very-high-glucose diet) study [19]. Another objective of this study was to investigate the impact of very-high-fructose intake, which has been found to alter lipid metabolism [20–23], on cholesterol homeostasis.

Visceral and liver fat contents were measured with magnetic resonance (MR) imaging and ^1^H-MR spectroscopy, respectively. To estimate cholesterol absorption and synthesis, we measured the serum concentrations of lathosterol, lanosterol, desmosterol (cholesterol precursors, indicate endogenous cholesterol synthesis), campesterol, sitosterol (plant sterols, indicate intestinal cholesterol uptake), and cholestanol (5-*α* saturated derivative of cholesterol indicates intestinal cholesterol uptake) [24–26].

## 2. Methods

### 2.1. Study Design and Diet

We report on an exploratory, prospective, randomized, single-blinded, outpatient, intervention study (TUbingen FRuctose Or Glucose study) [19]. Inclusion criteria were age 20–50 years, body mass index 20–35 kg/m², physical health, and not more than one-hour sports per week. Exclusion criteria were pregnancy, any relevant illness, fructose intolerance, medication, metal implants, regular alcohol consumption ≥10 g/day, and claustrophobia. The participants received 150 g (600 kcal) of either fructose or glucose per day for four weeks. They were blinded to the type of intervention. The sugar was provided in identical plastic packs of 50 g and had to be dissolved in water (50 g sugar in 250 mL water). The participants were instructed to consume the sugar in addition to a balanced weight-maintaining diet (50% carbohydrates, 35% fat, and 15% protein). Fructose or glucose was ingested three times a day (morning, midday, evening) with the main meals. Dietary counseling was provided by a trained dietitian according to the guidelines of the German Society of Nutrition. We aimed to assess compliance with the dietary prescription by close telephone contact. The participants were instructed to immediately inform the investigators in case of problems with the intake of fructose or glucose. For that, they were provided a calling card. Furthermore, compliance was evaluated by interview at visits 1 and 2. In addition, the subjects were asked to fill out food intake records on 3 days in each week of the study which were controlled and evaluated by a trained dietician using DGE PC software. Blood sampling, oral glucose tolerance testing, magnetic resonance imaging, and magnetic resonance spectroscopy were performed before and after dietary intervention. The study was approved by the local ethics committee and was conducted in accordance with the “Declaration of Helsinki.” Informed written consent was obtained from all participants. Data from the 20 participants who completed the study were included in the present analyses [19].

### 2.2. Laboratory Analyses

Total, HDL, and LDL cholesterol concentrations were measured with a standard colorimetric method on a Bayer analyzer (Bayer Health Care, Leverkusen, Germany). The serum noncholesterol sterols were measured using gas-liquid chromatography—mass spectrometry—selected ion monitoring (Hewlett Packard 5890) with an automatic injection system (Hewlett Packard Automatic Sampler 7673A) as previously described [27]. Blood glucose was determined using a bedside glucose analyzer based on a glucose-oxidase method (Yellow Springs Instruments, Yellow Springs, Colo). Insulin was analyzed by microparticle enzyme immunoassay (Abbott Laboratories, Tokyo, Japan).

### 2.3. Oral Glucose Tolerance Test

We performed standard 75 g oral gluose tolerance tests after a 10-h overnight fast. Venous plasma samples were obtained at 0, 30, 60, 90, and 120 min for determination of plasma glucose and insulin. Insulin sensitivity was estimated from the OGTT as proposed by Matsuda and DeFronzo: ISI_est_ = 10,000/√(Ins_mean_ × Gluc_mean_ × Ins_0_ × Gluc_0_) [28].

### 2.4. Quantitative Analysis of Visceral and Liver Fat

Visceral fat mass was measured with an axial T1-weighed fast spin echo technique with a 1.5 T whole-body imager (Magnetom Sonata; Siemens Medical Solutions) in the complete abdominal region, ranging from head of femur to head of humerus [29]. Liver fat was determined by localized proton magnetic resonance spectroscopy applying a single-voxel STEAM technique with short echo time (TE) as previously described [30, 31].

### 2.5. Statistical Analysis

The clinical and biochemical characteristics are presented as numbers and percentages and means ± standard errors of the means for categorical and continuous data, respectively. Ratios of the noncholesterol sterols to cholesterol (measured with gas-liquid chromatography) were calculated (see Table 2). The univariate relationships of the noncholesterol sterols with cholesterol, the relationships among the noncholesterol sterol to cholesterol ratios, and the relationships of the cholesterol subfractions and the noncholesterol sterol ratios with fat depots and insulin sensitivity were analyzed with linear regression models. The results are shown as Pearson correlation coefficients. Furthermore, we performed multivariate analysis for the associations of the cholesterol subfractions and the noncholesterol sterol to cholesterol ratios with fat depots and insulin sensitivity using Analysis of Covariance (ANCOVA). Alterations in the noncholesterol sterol to cholesterol ratios in response to fructose and glucose intervention were studied with the paired samples *t*-test (two-sided tests). ANCOVA was used to compare the changes in the noncholesterol sterol to cholesterol ratios (e.g., change in lathosterol to cholesterol ratio between baseline and followup examination) between the fructose and glucose intervention groups, with study group as the main factor and the metabolic parameter of interest at baseline (e.g., lathosterol to cholesterol ratio at baseline examination) as covariate (two-sided tests). To estimate the treatment effect, differences in least-square means and the corresponding 95% confidence intervals were calculated based on the ANCOVA models [32]. Data that were not normally distributed (Shapiro-Wilk *W* test) were transformed logarithmically (base-10). *P* values <0.05 were considered significant. The JMP statistical software package 7.0 (SAS Institute, Cary, NC, USA) was used.

## 3. Results

The baseline characteristics of the study participants are shown in Table 1. The mean ± standard error of the mean serum concentrations were 186 ± 5 mg/dL for cholesterol (GCMS), 0.241 ± 0.024 mg/dL for lathosterol, 0.091 ± 0.006 mg/dL for desmosterol, 0.058 ± 0.004 mg/dL for lanosterol, 0.295 ± 0.023 mg/dL for campesterol, 0.229 ± 0.018 mg/dL for sitosterol, and 0.320 ± 0.014 mg/dL for cholestanol.

The serum desmosterol and cholestanol levels were significantly related to cholesterol (*r* = 0.631, *P* = .002 and *r* = 0.615, *P* = .004, resp.). The lathosterol, desmosterol, and lanosterol to cholesterol ratios were also positively correlated (Table 3). In agreement, the ratios of campesterol and sitosterol to cholesterol showed a significant positive association (Table 3). Furthermore, the ratio of campesterol to cholesterol was significantly related to the ratio of cholestanol to cholesterol (Table 3). The ratio of lathosterol to cholesterol was inversely related to the ratio of campesterol to cholesterol (Table 3).

**Figure 1:**
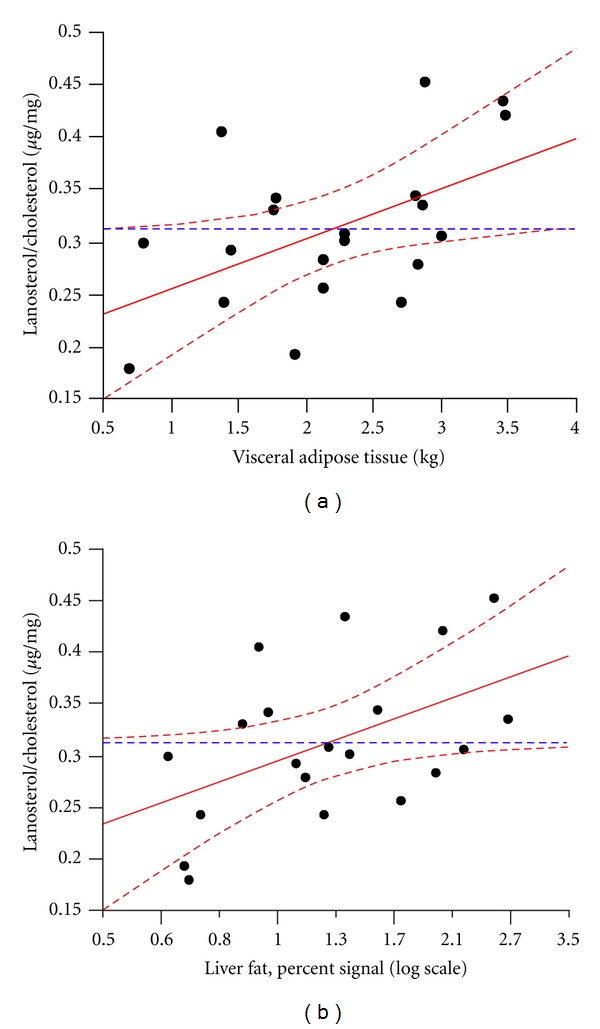
The associations of (a) visceral and (b) liver fat with the lanosterol to cholesterol ratio adjusted for sex, age, and body mass index.

High lanosterol and desmosterol to cholesterol ratios were significantly associated with increased visceral and liver fat content (Table 4). The association of the lanosterol to cholesterol ratio with visceral (*P* = .033) and liver fat (*P* = .044) was independent of sex, age, and body mass index (Figure 1). The cholesterol absorption markers were not significantly related to visceral and liver fat (Table 4). HDL cholesterol was inversely related to visceral fat and liver fat content whereas non-HDL cholesterol was positively correlated with visceral fat (Table 4). LDL cholesterol was not associated with fat depots (Table 4). The lathosterol to cholesterol ratio was inversely related to insulin sensitivity (Table 4).

The lathosterol to cholesterol ratio significantly decreased in response to very-high-fructose diet but not in response to very-high-glucose diet with the difference between interventions reaching statistical significance (Table 5). In agreement, there was a significant treatment effect for the alterations of the lanosterol to cholesterol ratio (Table 5). No changes or treatment effects were found for the desmosterol to cholesterol ratio and the absorption marker to cholesterol ratios (Table 5).

## 4. Discussion

We found that the ratios of desmosterol and lanosterol to cholesterol, which indicate endogenous cholesterol synthesis, were positively associated with visceral and liver fat content in persons at relatively low metabolic risk. The relationships of the lanosterol to cholesterol ratio with visceral and liver fat were independent of obesity. Furthermore, high ratio of lathosterol to cholesterol, also an indicator of cholesterol synthesis, was related to lower insulin sensitivity.

Our observations fit well in the context of the previously published studies in the field. High body mass index, insulin resistance, and type 2 diabetes have been independently associated with high cholesterol synthesis [6–11]. Visceral obesity and just recently fatty liver were also related to increased synthesis of cholesterol [12, 13, 18]. Our study extends these findings in the sense that we report on a cohort of healthy people. Furthermore, we have also measured the cholesterol synthesis marker lanosterol which was obviously the noncholesterol sterol most strongly related to visceral and liver fat content. We did not find significant associations of cholesterol absorption with visceral or liver fat. This may suggest that fat distribution and ectopic fat deposition in the liver primarily affect cholesterol synthesis. In this respect, it seems noteworthy that visceral fat was not significantly related to the sitosterol to cholesterol ratio in a recent study either [13]. 

Why is liver fat content positively correlated with cholesterol synthesis? The most important regulator of cellular cholesterol synthesis is the sterol regulatory element-binding protein 2 (SREBP2), a membrane-bound transcription factor [33, 34]. This transcription factor is highly expressed in the liver and interestingly, its activity is increased in subjects with high liver fat [35]. However, the exact mechanisms accounting for the activation of SREBP2 in subjects with high liver fat seem poorly understood. 

Since cholesterol homeostasis is obviously associated with liver fat content, the following question arises: might pharmacological interventions targeting cholesterol homeostasis have an impact on hepatic lipid content? A well-performed study by Szendroedi et al. found that even high-dose simvastatin treatment has no direct effects on liver fat content in people with type 2 diabetes [36]. In contrast, the cholesterol absorption inhibitor ezetimibe was found to increase the reduction of liver fat in obese subjects on a weight-loss diet [37]. Whether the use of plant sterols and stanols, which similarly act as inhibitors of cholesterol absorption [38], will help to reduce hepatic steatosis remains to be investigated.

We also studied the effects of very high-fructose and very-high-glucose diets on cholesterol absorption and synthesis. High-fructose diet has been implicated in the pathogenesis of the metabolic syndrome, fatty liver, and type 2 diabetes [20–23]. Moreover, a recent study suggested that high-fructose diet was associated with increased plasma concentrations of LDL cholesterol, small dense LDL, and oxidized LDL [23]. The effect of high-fructose diet on cholesterol homostasis has not been investigated so far. According to the present findings, fructose compared with glucose appears to less strongly stimulate cholesterol synthesis. This novel observation may be explained by the fact that fructose does not provoke endogenous secretion of insulin [20], which is considered to be an important regulator of cholesterol synthesis [39]. Alternatively, the treatment effect for cholesterol synthesis may result from the significant weight gain in the glucose intervention group (+1.7 kg) which was not observed in the fructose intervention group (+0.2 kg) [19].

Consistent with earlier work [12, 13, 40, 41], the serum HDL cholesterol concentration was significantly decreased in subjects with high visceral and liver fat content in the present cohort. Hence, increased visceral and liver fat content may indicate early disturbance of lipid metabolism in healthy people. It is also in agreement with a recent trial that the serum total and LDL cholesterol concentrations were not significantly related to visceral and liver fat content in our cohort of healthy individuals [12]. Hoenig et al. even showed that low-density lipoprotein cholesterol was inversely correlated with the abdominal visceral fat area in subjects with established vascular disease [13]. The authors discussed that their finding could explain the loss of the relationship between LDL cholesterol and cardiovascular events in the obese and support the use of non-HDL cholesterol instead of LDL cholesterol as the primary therapeutic target for lipid lowering therapy [13, 42]. Our data may support this view considering that we observed a positive correlation of non-HDL cholesterol with visceral fat. 

Finally, our data confirm that intestinal cholesterol absorption and endogenous cholesterol synthesis are interrelated considering the significant inverse association between the ratios of lathosterol and campesterol to cholesterol [7, 24].

The sample size of our study is relatively low. We cannot, therefore, rule out that a significant association of visceral and liver fat content with the cholesterol absorption markers could be observed in a larger cohort of healthy individuals. To compensate for this drawback, we used very precise and stringently validated analytical procedures for the quantification of the noncholesterol sterols and the fat depots. The serum concentrations of the noncholesterol sterols were measured using a highly sensitive and specific gas-liquid chromatography method. Visceral and liver fat content were quantified using magnetic resonance imaging and magnetic resonance spectroscopy, respectively. We also want to highlight that the sample size of our cohort was similar or even larger compared with previous highly recognized studies fructose intervention studies [21]. 

In conclusion, we found an independent association of visceral and liver fat content with cholesterol synthesis in healthy humans. Moreover, we were able to show for the first time that cholesterol synthesis is dependent on fructose/glucose intake. Studies investigating whether marked alterations of liver fat content will have an impact on cholesterol homeostasis are encouraged.

## Figures and Tables

**Table 1:** Baseline characteristics of the study participants.

	Baseline examination
Males/females, *n*	12/8
Age, years	30.5 ± 2.0
Body mass index, kg/m²	25.9 ± 0.5
Waist, cm	85 ± 2
Visceral fat, kg	2.2 ± 0.2
Liver fat, % signal	1.5 ± 0.2
Systolic blood pressure, mmHg	117 ± 3
Diastolic blood pressure, mmHg	77 ± 2
Total cholesterol, mg/dL	175 ± 5
LDL cholesterol, mg/dL	106 ± 5
HDL cholesterol, mg/dL	54 ± 2
VLDL cholesterol, mg/dL	15 ± 2
Non-HDL cholesterol, mg/dL	122 ± 5
Fasting glucose, mmol/L	4.86 ± 0.06
Fasting insulin, pmol/L	48 ± 7
Insulin sensitivity Matsuda, arbitrary units	17.6 ± 2.1

Values are numbers and percentages and means with standard errors of the means for categorical and continuous data, respectively.

**Table 2:** Serum levels of the noncholesterol sterol to cholesterol ratios at baseline.

Lathosterol/cholesterol, *μ*g/mg	1.28 ± 0.11
Desmosterol/cholesterol, *μ*g/mg	0.49 ± 0.02
Lanosterol/cholesterol, *μ*g/mg	0.31 ± 0.02
Campesterol/cholesterol, *μ*g/mg	1.58 ± 0.11
Sitosterol/cholesterol, *μ*g/mg	1.22 ± 0.09
Cholestanol/cholesterol, *μ*g/mg	1.72 ± 0.06

Values are means with standard errors of the means.

**Table 3:** Univariate correlations among the noncholesterol sterol to cholesterol ratios.

	Lathosterol/cholesterol	Desmosterol/cholesterol	Lanosterol/cholesterol	Campesterol/cholesterol	Sitosterol/cholesterol	Cholestanol/cholesterol
Lathosterol/cholesterol	—	0.572^†^	0.489^‡^	−0.493^‡^	−0.378	−0.378
Desmosterol/cholesterol	0.572^†^	—	0.607^†^	−0.280	−0.173	−0.357
Lanosterol/cholesterol	0.489^‡^	0.607^†^	—	−0.059	0.000	−0.036
Campesterol/cholesterol	−0.493^‡^	−0.280	−0.059	—	0.880*	0.486^‡^
Sitosterol/cholesterol	−0.378	−0.173	0.000	0.880*	—	0.375
Cholestanol/cholesterol	−0.378	−0.357	−0.036	0.486^‡^	0.375	—

Values are Pearson correlation coefficients calculated with linear regression; *P < 0.001, ^†^ *P* < 0.01, ^‡^ *P* < 0.05.

**Table 4:** Univariate correlations of total cholesterol, cholesterol subfractions, and the noncholesterol sterol to cholesterol ratios with liver fat, visceral fat, and insulin sensitivity.

	Liver fat		Visceral fat		Insulin sensitivity	
	*r*	*P*	*r*	*P*	*r*	*P*
Total cholesterol	0.194	0.384	0.243	0.303	0.082	0.730
LDL cholesterol	0.386	0.145	0.363	0.116	−0.049	0.839
HDL cholesterol	−0.545	0.011	−0.593	0.006	0.045	0.852
VLDL cholesterol	0.035	0.884	0.254	0.279	0.280	0.231
Non-HDL cholesterol	0.386	0.093	0.452	0.046	0.066	0.783
Lathosterol/cholesterol	0.107	0.636	0.404	0.078	−0.500	0.025
Desmosterol/cholesterol	0.548	0.027	0.541	0.014	−0.259	0.271
Lanosterol/cholesterol	0.642	0.004	0.629	0.003	−0.364	0.114
Campesterol/cholesterol	−0.005	0.710	−0.253	0.282	0.335	0.149
Sitosterol/cholesterol	0.040	0.803	−0.279	0.234	0.200	0.397
Cholestanol/cholesterol	0.008	0.974	0.060	0.800	0.325	0.163

*r* Pearson correlation coefficients and *P* values calculated with linear regression (liver fat was transformed logarithmically for calculation of the *P* values).

**Table 5:** Changes in the noncholesterol sterol to cholesterol ratios in response to high-fructose or high-glucose diet.

	Fructose intervention group			Glucose intervention group			Fructose versus glucose		
	Baseline	Change	P*	Baseline	Change	P*	ΔLSM	95% CI	*P* ^†^
Lathosterol/cholesterol, *μ*g/mg	1.16 ± 0.11	−0.20 ± 0.06	0.006	1.40 ± 0.20	−0.08 ± 0.18	0.659	−0.28	−0.53 to −0.04	0.027
Desmosterol/cholesterol, *μ*g/mg	0.45 ± 0.03	0.01 ± 0.02	0.675	0.52 ± 0.04	−0.02 ± 0.04	0.638	−0.01	−0.08 to 0.07	0.809
Lanosterol/cholesterol, *μ*g/mg	0.30 ± 0.03	−0.03 ± 0.03	0.332	0.32 ± 0.02	0.03 ± 0.04	0.555	−0.08	−0.16 to 0.00	0.040
Campesterol/cholesterol, *μ*g/mg	1.74 ± 0.13	−0.07 ± 0.07	0.337	1.42 ± 0.17	0.05 ± 0.15	0.746	−0.10	−0.47 to 0.27	0.569
Sitosterol/cholesterol, *μ*g/mg	1.28 ± 0.09	−0.07 ± 0.05	0.165	1.16 ± 0.15	0.01 ± 0.09	0.889	−0.06	−0.26 to 0.14	0.562
Cholestanol/cholesterol, *μ*g/mg	1.82 ± 0.06	−0.10 ± 0.10	0.348	1.61 ± 0.09	−0.07 ± 0.06	0.307	−0.04	−0.32 to 0.25	0.796

Values are means ± standard errors of the means; change: absolute difference between visits 1 and 2; fructose versus glucose: treatment effect of fructose intervention compared to glucose intervention; ΔLSM: difference in least squares means between fructose and glucose intervention (calculated with Analysis of Covariance with correction for baseline values); CI: confidence interval; *P value for change between visits 1 and 2 calculated with paired samples * t*-test (two-sided); ^†^ *P* value for difference in change between fructose and glucose intervention (calculated with Analysis of Covariance with correction for baseline values, two-sided).
